# Bedding types affect pubertal progression in rat

**DOI:** 10.1186/1687-9856-2015-S1-P96

**Published:** 2015-04-28

**Authors:** Byung-Ho Kang, Ja Hyang Cho, Won Seok Lee, Mun Suk Park, Kye Shik Shim

**Affiliations:** 1 Dept. Pediatrics/Endo, Kyung Hee University Hospital, Seoul, South Korea; 2 Kyung Hee University Hospital, Seoul, South Korea

## Aims

Recently, many experimental animal studies have demonstrated several adverse effects of endocrine disrupting agent, such as reduced reproductive behavior, altered estrous cycle, and decreased slow-wave sleep.

The purpose of this study was to compare the pubertal progression in wild type female rats according to different bedding types.

## Method

Twenty female Sprague Dawley rat (SD rat) were randomly divided into two groups according to their bedding types. The 1^st^ group was raised in wood shaving bedding as a control and 2^nd^ one was in corncob bedding as an endocrine disrupting agent.

Each group was checked daily for the first day of vaginal opening, and their vaginal smears were collected to determine estrous cyclicity after vaginal opening. The interval between vaginal opening and the first normal estrous cycle was recorded for determining sexual maturation.

The phases of normal estrous cycles were as follows, proestrus, estrus, metestrus and diestrus.

We compared the proportion of normal estrous cycles in each group.

## Result

Vaginal opening was shown early in corncob bedding, but there was no significant difference in the day of the first estrous cycle between 2 groups. Therefore, the periods between vaginal opening and the first estrous cycle were prolonged in that.

The proportion of normal estrous cycles was 80% in 1st and 60% in 2nd group.

In corncob bedding, the number of proestrus and estrus phases was significantly decreased and that of diestrus phases was increased.

## Conclusion

The onset of vaginal opening was earlier, and the irregularity of estrous cycles was increased in corncob bedding groups.

Endocrine disrupting agents in corncob bedding were considered to be associated with early vaginal opening and the irregularity of estrous cycles.

Therefore, the bedding type can be an affecting factor in pubertal progression in rodents.

